# Parallel Lemniscal and Non-Lemniscal Sources Control Auditory Responses in the Orbitofrontal Cortex (OFC)

**DOI:** 10.1523/ENEURO.0121-20.2020

**Published:** 2020-10-07

**Authors:** Hemant K. Srivastava, Sharba Bandyopadhyay

**Affiliations:** 1 Advanced Technology Development Centre, Indian Institute of Technology Kharagpur, Kharagpur 721302, India; 2 Department of Electronics and Electrical Communication Engineering, Indian Institute of Technology Kharagpur, Kharagpur 721302, India

**Keywords:** deviant detection, lemniscal non-lemniscal auditory pathways, persistent activity, stimulus history dependence, stimulus outcome association

## Abstract

The orbitofrontal cortex (OFC) controls flexible behavior through stimulus value updating based on stimulus outcome associations, allowing seamless navigation in dynamic sensory environments with changing contingencies. Sensory cue driven responses, primarily studied through behavior, exist in the OFC. However, OFC neurons’ sensory response properties, particularly auditory, are unknown in the mouse, a genetically tractable animal. We show that mouse OFC single neurons have unique auditory response properties showing pure oddball detection and long timescales of adaptation resulting in stimulus-history dependence. Further, we show that OFC auditory responses are shaped by two parallel sources in the auditory thalamus, lemniscal and non-lemniscal. The latter underlies a large component of the observed oddball detection and additionally controls persistent activity in the OFC through the amygdala. The deviant selectivity can serve as a signal for important changes in the auditory environment. Such signals, if coupled with persistent activity, obtained by disinhibitory control from the non-lemniscal auditory thalamus or amygdala, will allow for associations with a delayed outcome related signal, like reward prediction error, and potentially forms the basis of updating stimulus outcome associations in the OFC. Thus, the baseline sensory responses allow the behavioral requirement-based response modification through relevant inputs from other structures related to reward, punishment, or memory. Thus, alterations in these responses in neurologic disorders can lead to behavioral deficits.

## Significance Statement

Orbitofrontal cortex (OFC) has been shown to influence the stimulus representation in the sensory cortices allowing them to adjust to the changing contingencies in the environment, but how OFC itself receives and engages with the incoming stimulus is poorly understood. The response properties of the OFC neurons from a sensory perspective, independent of behavioral state and other cognitive processes, are not known. We show that OFC robustly responds to auditory stimulation with strong context dependence and selectivity to oddball or deviant stimuli. We also show that both lemniscal and non-lemniscal pathways, both at cortical and subcortical levels, differentially contribute to auditory responses in the OFC.

## Introduction

The orbitofrontal cortex (OFC), a part of the prefrontal cortex (PFC), is involved in flexible behavior (Miller and Cohen, 2001; Wallis, 2007; Fritz et al., 2010) by encoding specific stimulus–outcome or action–outcome expectancies as well as by dynamically revaluing such expectancies based on behavioral demands and motivational states (Delamater, 2007; Rudebeck et al., 2008; Wilson et al., 2014; Fresno et al., 2019). Specific OFC circuits can control specific aspects of flexible behavior and multiple reinforcement learning processes (Lee et al., 2012; Groman et al., 2019). For OFC neurons to encode the sensory attributes and subjective value of outcomes associated with external stimuli (Schoenbaum and Roesch, 2005; Delamater, 2007; Ostlund and Balleine, 2007), it requires sensory inputs to begin with. It is known that sensory stimulus-evoked signals in the OFC can distinguish between appetitive and aversive outcomes (Morrison and Salzman, 2011) associated with the stimuli. Further, the OFC can also influence sensory processing by modulating neuronal receptive fields in early sensory cortices, particularly the auditory cortex (AC; Winkowski et al., 2013).

To understand how specific stimulus outcome associations are created and how the stimulus-evoked OFC responses may influence sensory representation, it is crucial to delve into the origins of sensory inputs and sensory response properties of the OFC. In the case of auditory stimuli, the pathways involved and their contribution to auditory responses in the OFC are not known. What aspects of information in the ongoing auditory environment, how and in what form reaches the OFC would determine how mechanistically stimulus-outcome expectancies or values would get computed or updated. We, therefore, as a first step, consider the auditory evoked responses of OFC neurons, from a sensory perspective and attempt to decipher the crucial components of the auditory pathway involved in shaping auditory responses and their properties in the OFC.

Although mouse OFC single neurons have been shown to respond to a variety of sounds (Winkowski et al., 2018), it is unclear how selective the responses are and how they change under different sensory contexts. With single-unit recordings in mice, both awake and anesthetized, we show that auditory responses in the OFC are strongly context dependent with long timescale history dependence. We also show that the OFC neurons respond only to a change or deviant in the stimulus stream and cease to respond to any repeating stimuli from the first instant of repetition. We call this response characteristic as pure oddball detection. We also show that these response properties are different from the AC, possibly arising either within the local circuits of the OFC or regions other than AC. Investigation of anatomic and functional sources of inputs show that both the lemniscal and non-lemniscal pathways at the cortical and subcortical levels shape auditory responses in the OFC. With pharmacological inactivation experiments, the contributions of multiple auditory cortical and subcortical areas in OFC’s auditory responses were assessed. In the AC, the dorsal region (AuD), with the most projections to the OFC, surprisingly did not contribute to auditory responses of the OFC. In contrast, the other higher-order non-lemniscal ventral auditory area (AuV; Sacco and Sacchetti, 2010), was found to be the main source of auditory evoked excitatory drive to the OFC. The primary AC (A1) contributed to temporal response properties in the OFC. Further, considering the auditory thalamic (medial geniculate body; MGB) sources showed that non-lemniscal AuV’s contribution to OFC responses originates from the lemniscal MGBv through its direct projections to AuV (Ohga et al., 2018). The non-lemniscal, polymodal medial division of MGB (MGBm; Weinberger, 2011; Lee, 2015) inactivation, however, caused the OFC auditory responses to become persistent, similar to frontal cortex responses during working memory dependent tasks (Fuster and Alexander, 1971; Funahashi et al., 1989; Schoenbaum and Setlow, 2001). Thus, the MGBm is a source that causes auditory driven long-lasting inhibition in the OFC. Inactivation of basolateral amygdala (BLA), providing inhibitory inputs (Dilgen et al., 2013; McGarry and Carter, 2016; Lichtenberg et al., 2017) to the OFC and known to receive MGBm inputs via the lateral amygdala (LA; Ledoux, 2000; Woodson et al., 2000), showed similar emergence of auditory driven persistent activity. Further, the same pathway contributed substantially to the strength of deviant selectivity in the OFC. We suggest that the feedforward inhibition (Dilgen et al., 2013) from BLA to OFC and parallel MGBm to LA inhibition (Woodson et al., 2000) transmitted to OFC via BLA allow for two independent controls to generate persistent activity in the OFC required for stimulus outcome associations.

## Materials and Methods

### Animals

All animal experiments were approved by Institutional Animal Ethics Committee (IAEC) of Indian Institute of Technology Kharagpur. Animals were reared under a 12/12 h light/dark cycle and maintained at a temperature of 22–25°C and had access to food and water *ad libitum*. C57BL/6 mice of either sex, postnatal (P) age between P-25 and P-45, were used for the experiments (Table 1). Data acquired in pharmacological block experiments before blocking were also included in the analysis of OFC responses.

### Animal preparation

#### Anesthetized recordings

Animals were anesthetized using isoflurane (5% for induction and around 1–1.5% for maintenance). Body temperature was maintained at 39°C by placing the animal on a heating plate. A small incision was made to expose the skull and a metal plate was attached on to the skull to head fix the animal. Once head fixed, a small (∼2 mm in diameter) craniotomy was performed to remove the skull over the recording site. For OFC, the stereotaxic coordinates used were AP = +2.5 mm, ML = 1 mm from the bregma, DV = 1.8 mm (Paxinos and Franklin, 2013) from the brain surface. For AC, the recording site was identified based on the vasculature (Sawatari et al., 2011). All recordings were performed with a microelectrode array (MEA) with a 4 × 4 grid (125 μm between rows and columns) of epoxy-coated tungsten electrodes (MicroProbes, impedance ∼3–5 MΩ).

#### Awake head-fixed recordings

As in the anesthetized case, a similar but smaller craniotomy (∼1 mm in diameter) was performed and the electrodes were advanced into the recording site and the held fixed on the skull using Metabond (C & B superbond). A titanium plate was also fixed to the skull (posterior to the electrodes) with Metabond to head-fix the animal during the experiments. Animals were allowed to recover for 5 d and then were habituated with the recording setup for 30 min for 3 d before data collection. Data collection lasted for <7 d, with ∼1-h-long sessions every day. Units collected on each day from the recording electrodes were considered as separate units.

#### Stimulus

All acoustic stimulation was presented from the right side, contralateral to the recording site. Initially, noise bursts [6- to 48-kHz bandwidth, 50 ms, 5-s intertrial interval (ITI), of multiple intensities, 40- to 0-dB attenuation, in 10-dB steps; 0-dB attenuation corresponds to ∼90-dB SPL for tones] were used to obtain threshold sound level for noise. Next single units were characterized with pure tones (50 ms, 6–48 kHz, 1/2 octave apart, 70- to 80-dB SPL, depending on noise threshold, 5 s apart, except mentioned otherwise) to obtain tuning and the best frequencies (BFs) at the chosen sound level. Next response to a pair of oddball stimulus set was collected. The oddball stimulus consisted of a standard token (S; 50 ms, either a noise token or a pure tone) and deviant token (D; 50 ms, either a pure tone or a noise token, respectively, for noise-tone, NT or TN, oddball; S and D were both pure tones in case of tone-tone, TT, oddball). The S-D stream had 15 tokens usually presented at 4 Hz or 3.3 Hz; all the tokens were S tokens except the eighth token which was the D token. In the second of the pair of the oddball stimulus set, the S and D tokens were swapped. Each oddball set was repeated 20–30 times with a gap of >5 s between each repetition. In some animals, the total number of tokens was changed to 20, and the location of deviant was also changed to 12th. In these animals, the response profile was not different from the usual 15 tokens case. All sound tokens presented in all kinds of stimuli had 5-ms rise and fall times. Since recordings were with MEAs with 16 electrodes, the pure tone frequency was chosen based on the tuning of the majority of simultaneously recorded neurons, such that the chosen tone frequency was within the receptive field of most neurons. The stimuli were generated using custom-written software in MATLAB (MathWorks) and presented with Tucker Davis Technologies (TDT) ES1 speakers (driven with TDT ED1 drivers) after generation with a TDT RX6 processor and attenuated using a TDT PA5. The speaker was placed 10 cm away from the contralateral ear.

### Electrophysiology

#### Anesthetized

The MEA was slowly advanced into the recording site with the help of a manipulator (MP-225, Sutter). The electrodes were allowed to settle and stabilize for ∼30 min before the data were acquired. Data were collected using custom-written software (MATLAB), through a unity gain headstage (16 channels, Plexon HST 16o25) amplifier, followed by a preamplifer (PB3, Plexon, 1000×). Wide-band neural signals (0.7 Hz to 8 kHz) as well as a parallel set of 16 channels with spike signals (150 Hz to 8 kHz) were stored after digitizing at 20 kHz using a A/D board (National Instruments). Off-line analysis was performed with stored data. At the end of the experiment, the animal’s brain was harvested for *post hoc* examination of the recording site.

#### Awake

For awake recordings, the animal was placed in a small tube with head protruding out and fixed using the titanium plate implanted during surgery. The rest of the procedure was similar to the anesthetized recordings.

#### Anatomy

Nine animals were injected with 200 nl of green retrobeads (Lumafluor) into OFC and three of them were also injected with 100 nl of anterograde tracing *AAV.CB7.CI.mCherry* in MGBv using Nanoject II. After 14 d of injection, the animals were transcardially perfused with 20-ml PBS followed by 20 ml of 4% paraformaldehyde and brain was harvested and kept in 4% paraformaldehyde overnight. 100-μm-thick brain sections were cut using a vibratome (Leica VT1000S), mounted on a glass slide with fluomount cover slip, and observed under a fluorescence microscope (Leica DM2500).

#### Electrophysiology with pharmacological inactivation

A small burr hole was made on the skull over the area to be inactivated, ipsilateral (or both sides as mentioned) to the recording site. A Hamilton syringe (7000 series) loaded with the 200 nl of GABA agonists (5 μg/μl muscimol and 2 μg/μl baclofen) or equal volume saline was inserted and held via a cannula implanted on the skull with dental cement. Only after the syringe was positioned securely, the electrodes were inserted into the recording site as described above. For injecting the agonists/saline during the experiment, the Hamilton syringe was gently pressed/tapped multiple times over a 5- to 10-s period to release the entire volume. There was a waiting period of 30 min for the agonists to have their effect before the next data set was acquired. SR101 was added to the mixture of agonists or saline for *post hoc* confirmation of the target site and spread of the injection. Different divisions of AC were identified and marked based on the vasculature. For MGB (Slater et al., 2019) and BLA (Luna and Morozov, 2012) following stereotaxic coordinates were used: MGB: AP = −3.27 mm, ML = 2.0 mm from bregma, −3.0 mm from the brain surface; and BLA: AP = −1.3 mm, ML = 3.2 mm from bregma, 3.8 from the brain surface.

### Data analysis

Spike Sorting was done offline in custom-written MATLAB software. Data were notch filtered (Butterworth fourth order) to reject any remnant power supply 50-Hz oscillations. Spiking activity was obtained directly from the spike channels of the PBX3 preamp. Waveform fluctuations above 3.5–4 SDs (usually 4) from the baseline were isolated and based on shapes, spike waveforms were clustered into different groups. The timing of spikes with respect to data collection onset (and hence also stimulus presentation) was extracted for each spike shape (single unit) for further analysis. A single unit was considered as responsive if the spike rate within 400 ms (200 ms in case of awake condition) of stimulus presentation was significantly different from the baseline (300 ms preceding the stimulus, *t* test, α = 0.05). Response latency was calculated as the time at which the spike rate in the average peristimulus time histogram (PSTH; 20-ms bins) was maximum.

#### Narrow tuning and calculation of BF

Tuning of neurons was considered to be narrow (well tuned) or bimodal and/or broad. The frequency corresponding to the maximum response rate out of the seven frequencies presented to narrowly-tuned units (below) was considered the BF of the unit. Those units were narrow -tuned whose average response to frequencies other than one octave around BF was 2 SDs (variability in response rate at BF) below the response at BF.

#### Spatial BF variability

The SD of BFs of all the simultaneously acquired units (only narrowly tuned, above) was normalized by the product of area accommodating these responding units and the number of units:

(1) $$Spatial\;BF\;variability=\frac{std\left(BFs\;of\;simultneously\;acquired\;units\right)}{area\;X\;number\;of\;units}.$$

For simulating a distribution of completely heterogeneous BFs, each unit was randomly assigned a BF (uniformly over the seven frequencies used) and BF variability was calculated with 1000 bootstraps.

#### CSI calculation

For common selectivity index (CSI) calculation, those units were included which responded to at least one of the four stimulus tokens, first of each of the S tokens in the oddball pair S_X_ and S_XS_ (XS being the swap of the X oddball), and the deviant tokens, D_X_ and D_XS_. CSI was calculated as per the following equation (Ulanovsky et al., 2003):

(2) $$CSI=\frac{\left(D_{X}+D_{XS}\right)-\left(S_{X}+S_{XS}\right)}{\left(D_{X}+D_{XS}\right)+\left(S_{X}+S_{XS}\right)},$$

where *S_i_*/*D_X_*_/_*_XS_* represents the mean rate response to those tokens, *S_i_* the *i^th^* token and *S_PT_* being the token preceding the *D* token. The rate responses in each case were computed based on the following windows: for S_1_, 100–400 ms from S_1_; for S_ALL_, S_2_ +100 ms, until deviant, entire length; for S_PT_, 100 ms from S_PT_ up to deviant; and for D, 100–400 ms from deviant.

When a sufficient sized population (at least 50 units) of paired data were not available (as in the case of tone-tone, TT oddball and in before versus after pharmacological inactivation experiments), deviant selectivity index (DSI) of each unit was calculated as follows:

(3) $$DSI=\frac{D_{X}-S_{X}}{D_{X}+S_{X}}.$$

#### Anatomy

The number of retrobeads was quantified using a threshold that was manually set for individual sections depending on the background intensity. The laminar demarcation was based on distance from pia, which was also corroborated in a subset with the MGBv projections observed with AAV.CB7.CI.mCherry. All beads within 300 μm from the pia were marked as layer 2–3, beads between 300 and 450 μm (or mCherry) were marked as layer four and beads below that were grouped into layer 5–6.

Demarcation of AuV, A1, and AuD to confirm correct injections through the spread of SR101 in blocking solution was through brain atlas (Paxinos and Franklin, 2013) and distance from rhinal fissure. Only those animals were included in the dataset whose spread was *post hoc* confirmed as above. Injections in MGBv and MGBm were targeted and *post hoc* confirmed through brain atlas (Paxinos and Franklin, 2013).

#### Response duration

A sliding response window of size 100 ms starting from the stimulus start, in 20-ms steps, was compared with the random 100-ms window in the baseline for significance. Consecutive significant bins with a time difference of <100 ms between them were joined together for the determination of response duration.

### Spike timing jitter

The spike timing jitter expressed as variability in the timing of individual spikes across trials was computed as the reliability (R_corr_) in spike timing which is a measure of similarity between pairs of individual spike trains (Schreiber et al., 2003). The trial wise individual spike trains binned at 1 ms was convolved with a Gaussian filter of σ = 2 ms, and then the coefficient of correlation was calculated between all pairs of trials using the MATLAB function *corrcoef*. Higher the R_corr_ value, less is the spike timing jitter.

#### Pairwise correlations

Pairwise correlations were calculated as correlation coefficient using the MATLAB function *corrcoef* between mean PSTHs (from stimulus start) of simultaneously recorded units.

## Results

### OFC neurons respond to sound with very long timescale dependence

**Figure 1.**
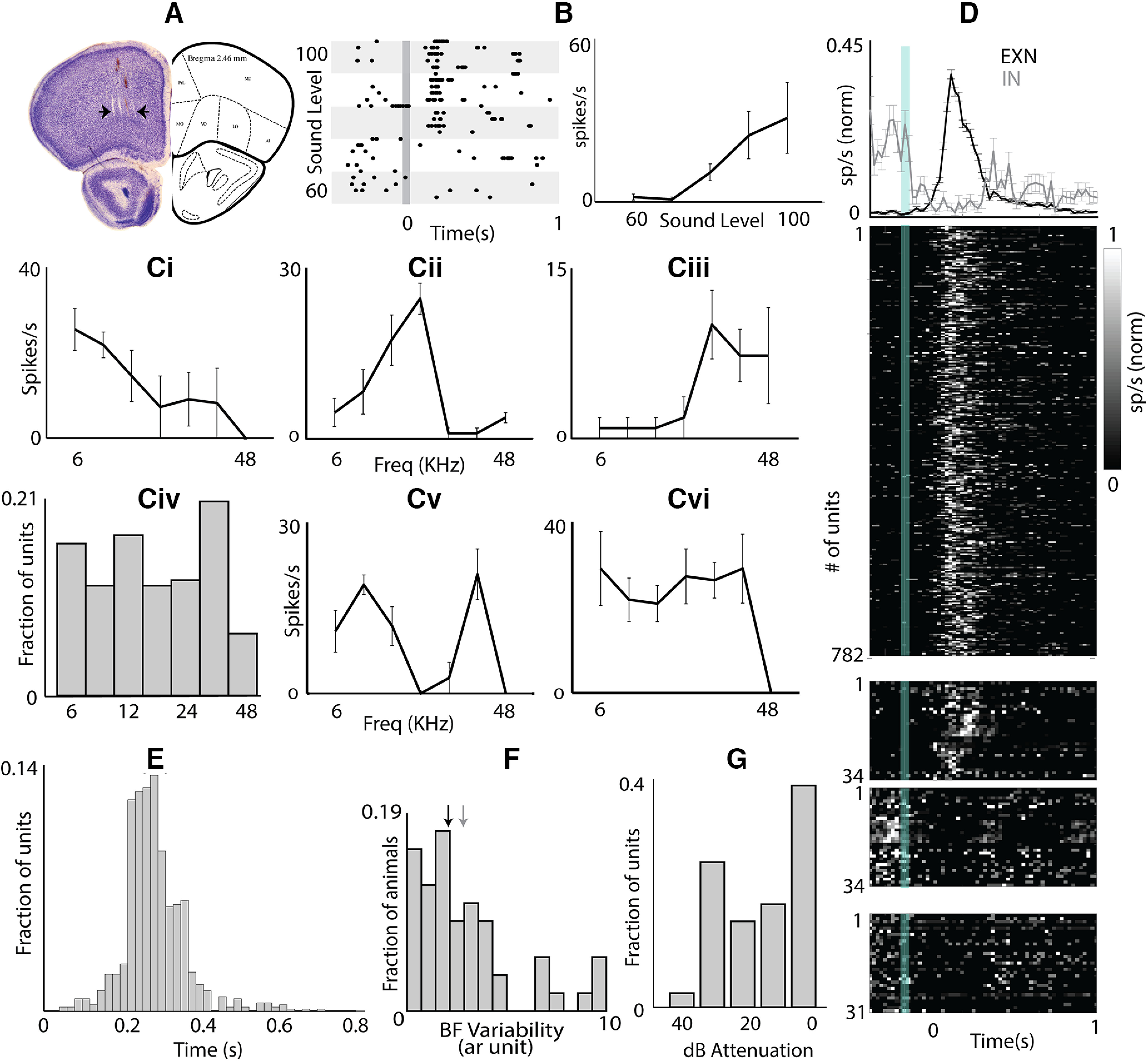
OFC responds to auditory stimulation. ***A***, Nissl stain of OFC section showing electrode tracks. Black arrows mark the medio-lateral extent of the electrode array. ***B***, An example unit showing responses to increasing intensities of broadband noise in raster plot (left) and rate-level curve (right). ***Ci–Ciii***, Tuning curves of three example units tuned to low (***Ci***), mid (***Cii***), and high (***Ciii***) frequencies. ***Civ***, Distribution of units tuned to different frequencies. ***Cv***, An example unit with bimodal tuning curve. ***Cvi***, An example unit with broad tuning curve. ***D***, top, Mean normalized PSTH ± SEM of all units showing excitation (black) and inhibition (gray) on auditory stimulation. Bottom, Individual unit’s PSTH (upper panel: units showing excitation, middle two panels: units showing excitation to some frequency and inhibition to some other frequency, lower panel: units showing inhibition). ***E***, Distribution of peak response latency to the BF. ***F***, Spatial BF variability. Black arrow marks the median of the distribution and gray arrow marks the median of the distribution of completely random BF organization. ***G***, Distribution of broad band noise thresholds; 0-dB attenuation corresponds to ∼90-dB SPL for a tone at 17 kHz.

To first obtain OFC single neuron responses to sounds, independent of behavioral state, active memory, and other cognitive processes, we recorded single units in the anesthetized mice. The recording sites were confirmed to be in the OFC (lateral and/or ventral) by *post hoc* Nissl staining of coronal sections (Fig. 1*A*). We found that neurons in the mouse OFC robustly responded to sound stimulation (Fig. 1*B*,*C*) with broadband noise and pure tones (also see Winkowski et al., 2018), with typically monotonic rate intensity functions with noise (Fig. 1*B*, right) and variety of BFs (Fig. 1 *Ci–Civ*) with narrow (Materials and Methods; 45% of the responding units) and bimodal to broad tuning (55% of the responding units; Fig. 1 *Cv*,*Cvi*). While most of the responding units increased their firing rate at BF (∼86%, 816/949 units), about ∼14% (133/949) of units reduced their firing rate at some frequency (Fig. 1*D*); 76 of the 133 units were excited at BF while the rest showed no excitatory responses. Notably, the suppression observed in all the 133 units (Fig. 1*D*), were all at either 6 or 8.5 kHz. The mean peak latency observed at the BF of units with excitatory responses was 284 ± 3 ms (Fig. 1*E*). The latency was substantially longer than what is seen in the mouse AC single-unit responses but similar to the late component of auditory responses (Chen et al., 2015), likely in the non-A1.

To check for the possible topographical organization of tuning based on the narrowly tuned units in the horizontal plane of the OFC, we considered the variability of BF of simultaneously recorded units. The distribution of calculated spatial BF variability in simultaneously recorded units (Eq. 1; Materials and Methods) across all recording locations was compared with a distribution expected with completely random BFs at each recording site (Materials and Methods; Fig. 1*F*, black arrow median of data, gray arrow median of the distribution with spatially random BFs). The two distributions were not significantly different (Kolmogorov–Smirnov test, *p* = 0.06). This suggests that the local organization is entirely random, indicating the absence of any BF based organization at the spatial scales (∼400 μm) of our recording along the horizontal plane in OFC. Distribution of level thresholds for broadband noise is shown in Figure 1*G*. The noise (6- to 48-kHz bandwidth) threshold in dB attenuation of our system corresponds to ∼20 dB above tone threshold. Thus, tones used in the study were played usually at an attenuation that is 10 dB above or at the noise threshold, which corresponds to 20–30 dB above the usual tone threshold in the 6- to 48-kHz range.

**Figure 2.**
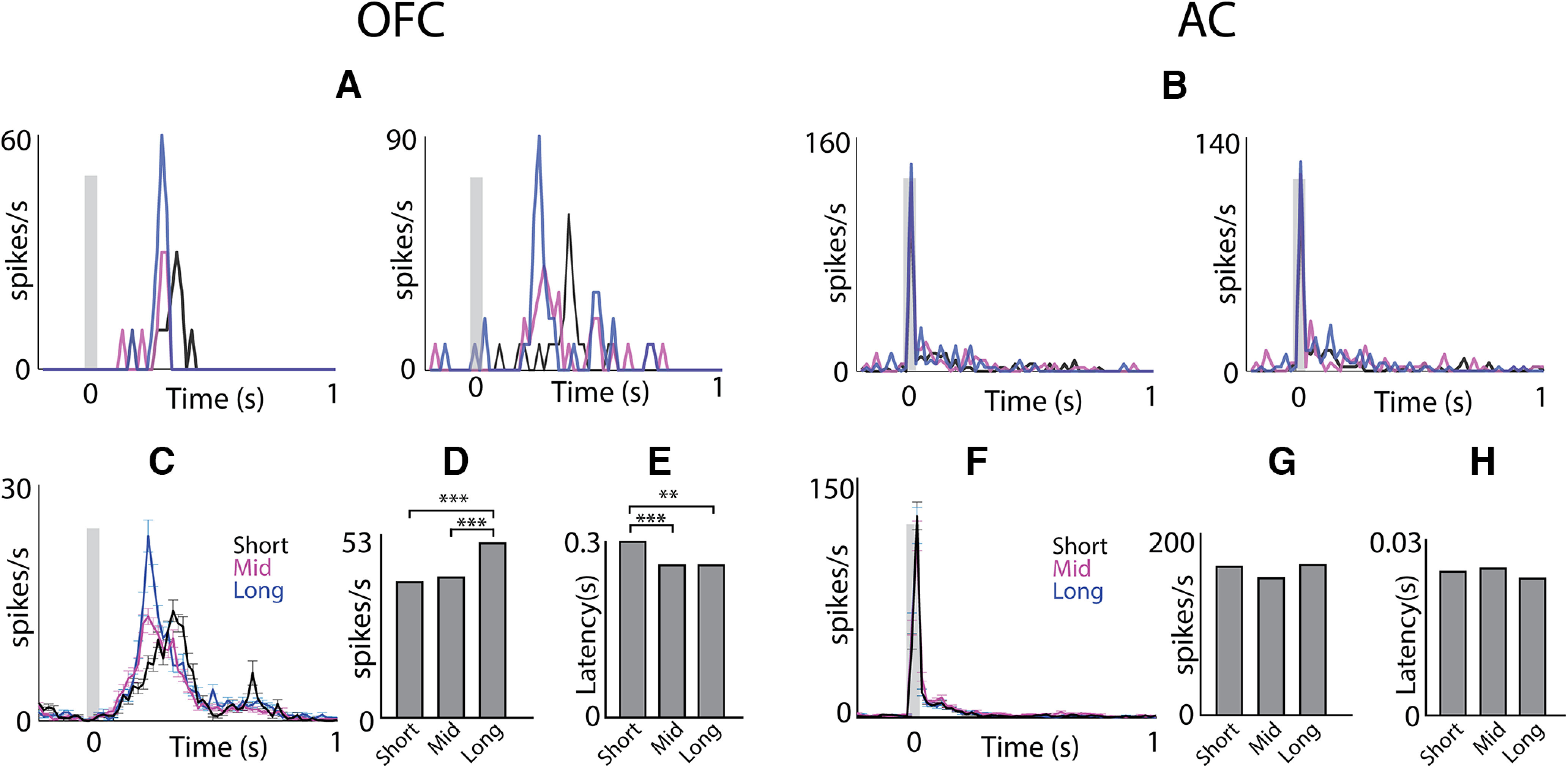
OFC neurons show long timescales of adaptation. ***A***, Psth of two example units in the OFC at different ITIs [black; <5 s (short), magenta; between 5 and 7 s (mid), blue; >7 s up to 11 s (long)]. ***B***, Similar to ***A*** in the AC. ***C***, Mean population PSTH ± SEM at different ITIs in the OFC. ***D***, Mean peak spike rate in the OFC. ***E***, Mean peak response latency in the OFC. ***F***, Mean population PSTH ± SEM at different ITIs in the AC. ***G***, Mean peak spike rate in the AC. ***H***, Mean peak response latency in the AC. **p < 0.01, ***p < 0.001.

To investigate the effects of adaptation and stimulus history, we recorded OFC responses to pure tone presentation with varying ITI with a short interval (1 and 3 s) less than 5 s, mid-interval ranging from 5 to 7 s and long interval ranging from more than 7 up to 11 s (Fig. 2*A*,*C*) and compared it to AC (Fig. 2*B*,*F*, both A1 and AuV; determined from *post hoc* Nissl stains with electrode tracks). We found that the response profiles of these three ITIs in the OFC were significantly different with one another (one-way ANOVA; Fig. 2*C*,*D*). The peak spike rate of the long ITI group was significantly different (one-way ANOVA, *p* < 0.001) from the mid and short ITIs (Fig. 2*D*), whereas the latency (Fig. 2*E*) of the short group was significantly different from the other two groups (one-way ANOVA *p* < 0.01 between short and long and *p* < 0.001 between short and mid). These results indicate that OFC neurons show very long (at least up to tens of seconds) timescales of stimulus history dependence that is reflected in the spike rate or latency of the response. Similar analyses of the AC neurons (Fig. 2*F–H*) show that neither the peak spike rates (Fig. 2*F*,*G*) nor the response latencies (Fig. 2*H*) were different in the three groups. Thus, OFC responses to auditory stimuli were found to have unprecedented temporal stimulus history dependence. Such remarkable dependence of sensory responses on long stimulus history, unlike in the sensory cortex, would be crucial in normal environments with continuously varying sensory inputs.

### Neurons in the OFC show high context dependence and pure oddball detection unlike in the AC

**Figure 3.**
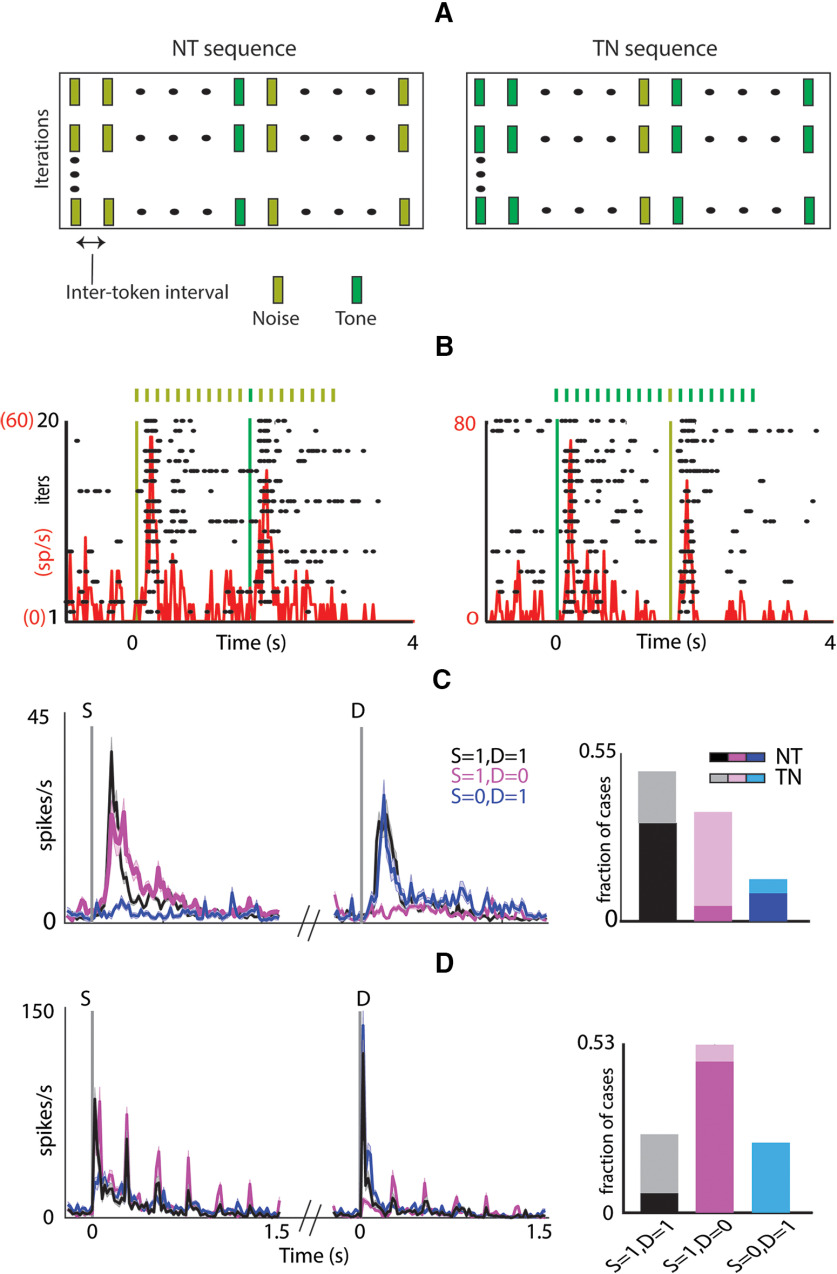
Pure oddball Detection in OFC unlike AC (i). ***A***, Oddball Stimuli showing NT (left) and TN (right) sequences. ***B***, An example unit showing auditory response (raster (black) and PSTH (red) to an oddball stimulus where noise is played as standard and tone as deviant (NT; left) and its swap (TN; right). The vertical lines mark the onset of standard and deviant (yellow for noise and green for tone). ***C***, left, Mean population PSTH ± SEM of units responding to both standard onset and deviant (S = 1, D = 1; black), responding only to standard onset (S = 1, D = 0; magenta) and responding only to deviant (S = 0, D = 1; blue). Right, Fraction of units belonging to different categories as described in ***B***. The darker shades show the fraction of units belonging to NT and lighter shades show fraction of units belonging to TN group. ***D***, Similar to ***C*** in the AC.

Given the long temporal dependencies, presumably because of strong and long-lasting adaptation, it becomes important to find the key aspects in streams of sounds to which neurons in the OFC respond. Since neurons in the OFC responded to both tones and noise (Fig. 1), we collected OFC responses to an oddball stimulus set with noise tokens as the standard (S) stimulus with a pure tone embedded in the stream as the D token (NT) and its swap (TN; Fig. 3*A*; Materials and Methods). We found that OFC neurons robustly responded to the D token (Fig. 3*B*). Typically, a strong onset response to the first of the standards (a deviant/change from the pre-stimulus silence) was seen, followed by a strong response to the D. This pure oddball detection characterized by responses to the first S token (S_1_) and D, followed by strong adaptation such that there is no response to the succeeding tokens, is not seen in AC. Thus, the responses in the OFC to oddball stimulation show strong and fast stimulus-specific adaptation (Taaseh et al., 2011; Nieto-Diego and Malmierca, 2016).

We obtained responses to oddball stimulus pairs (NT and TN) from 109 OFC units (10 animals, 218 cases; Table 2), where a unit was considered responding if it responded to at least one of the four stimulus tokens (S_1_ and D in each of the two in a pair; Materials and Methods). In 36% of the cases, units responded to both the S_1_ and the D token (Fig. 3*C*, black; Table 2), while in 31% cases, units responded to S_1_ but not D (Fig. 3*C*, magenta; Table 2). Further, 17% responded to only the D token (Fig. 3*C*, blue; Table 2) and the remaining 16% did not respond to either. In the last 16% cases, there was however a response to one of the stimulus tokens (S_1_ and/or D) in the corresponding swap oddball stimulus. Thus, although OFC neurons were found to be generally responsive to both, the tone and the noise when played as an individual token (Fig. 1), their response to the same sound token changed depending on the context of the stimuli (as in oddball stimulus). We find that when a neuron responded to a tone as S_1_ (Table 2), it was less likely to respond to the noise token as D (65/82: did not respond to noise as D and 17/82: responded to noise as D, *p* < 0.001, two proportion *z* test; Table 2). On the contrary, if a neuron responded to the noise token as S_1_ (Table 2), it was more likely to also respond to the tone token as D (62/65: responded to tone as D and 3/65: did not respond to tone as D, *p* < 0.001, two proportion *z* test; Table 2). Such context-specific selectivity is contrary to expectations, as a broadband noise as S is likely to adapt all frequency channels, thus masking the response to a tone as D embedded in a sequence of noise tokens. The above suggests that the OFC auditory circuitry is inherently more selective in detecting narrowband sounds in broadband background noise. However, when considering the response of the same unit to a stimulus token (noise or tone) as the S and as D, we find that out of 109 units, 75(17), 7(48), 8(16), and 19(28) units responded to the tone (noise) both as S and D, only as S, only as D, and neither, respectively (Table 3). The above suggests that responses to the tone were less context-sensitive than noise with more units responding to tones than to noise (tones: 75/109 and noise: 17/109, *p* < 0.001, two proportion *z* test) independent of their occurrence as S or D. This further corroborates the fact that the OFC neurons inherently are more responsive to tones and hence likely to narrowband tonal sounds like mouse vocalizations.

**Table 1:** Number of animals used by experiment

Experiment	Number of mice
OFC electrophysiology	27
AC electrophysiology	5
Anatomy	9
A1 inactivation (+ bilateral)	7 (+ 2)
AuV inactivation	5
AuD inactivation	3
MGBv inactivation	2
MGBm Inactivation	5
BLA inactivation	5
Awake OFC electrophysiology	5
Total	74

**Table 2:** Different response types to NT and TN stimuli in the OFC

218 cases	S_1_ = 1 and D = 1 cases	S_1_ = 1 and D = 0 cases	S_1_ = 0 and D = 1 cases
NT	62	03	21
TN	17	65	16
Total	79 (36%)	68 (31%)	37 (17%)

**Table 3:** OFC response types depending on the stimulus type and its location in the sequence

109 units	S_1_ = 1 andD = 1 (units)	S_1_ = 1 andD = 0 (units)	S_1_ = 0 andD = 1 (units)	S_1_ = 0 andD = 0 (units)
Tone as S_1_or D	75	07	08	19
Noise as S_1_or D	17	48	16	28

In contrast, we found nearly an opposite pattern of context dependence in the AC. Responses to oddball stimuli pairs (NT and TN) from a total of 62 units (51 in A1, 11 in AuV, 124 cases; Fig. 3*D*) were collected from the AC that responded to at least one of the four stimulus tokens (as above). In 22% of the cases, units responded to both S_1_ and D (Fig. 3*D*, black, bar plot; Table 4), while in 48% cases, units responded to S_1_ but not D (Fig. 3*D*, magenta, bar plot; Table 4). Further, 20% responded to only the D token (Fig. 3*D*, blue, bar plot; Table 4), and the remaining 10% did not respond to either. As expected from adaptation along frequency channels, when there was a response to the noise as S, very few units responded to the tone as D (7/61) and most were unresponsive to the tone (54/61), opposite of what was observed in the OFC. Considering responses of the same neuron to the tone or noise token in either of the two contexts (as S_1_ or D), 7(45), 20(16), 0(1), and 35(0) out of 62 AC units responded to tone (noise) as both S_1_ and D, only as S_1_, only as D and neither of the two, respectively (Table 5). In the AC, contrary to observations in the OFC, the tone responses independent of S and D were absent, while noise responses occurred almost independent of S and D (tones: 7/62 and noise: 45/62, *p* < 0.001, two proportion *z* test). There were many more cases in the AC when there were no responses to the tone either as S or as D. Such units were present in both A1 and AuV in similar proportions (30/51 and 5/11, NS). Although the choice of the tone frequency (Tf) for oddball sets (NT and TN) with respect to the BF of neurons were similar for OFC and AC (mean(|BF-Tf|) 1.3 octaves for OFC and 1.1 octaves for AC, not significant (NS), unpaired *t* test), the more number cases where there was no response to tones (as S or D) in AC could be because of narrower tuning profiles. To test this, we checked whether the units that did not respond to tones in the oddball stimulus responded to individual tone pip or not. We found that 27% (17/62) responded to both the stimuli, whereas 23% (14/62) units showed contextual modulation with 10 units responding only to oddball stimulus and 4 units responding only to tone pip. Remaining 50% of the units (31/62) did not respond in both the cases reflecting narrow tuning profile in the AC. This further corroborates with the finding that OFC neurons are more broadly tuned (Fig. 1) as compared with AC.

**Table 4:** Different responses types to NT and TN stimuli in the AC

124 cases	S_1_ = 1 and D = 1 cases	S_1_ = 1 and D = 0 cases	S_1_ = 0 and D = 1 cases
NT	07	54	0
TN	21	06	25
Total	28 (22 %)	60 (48 %)	25 (20%)

**Table 5:** AC response types depending on the stimulus type and its location in the sequence

62 units	S_1_ = 1 andD = 1 (units)	S_1_ = 1 andD = 0 (units)	S_1_ = 0 andD = 1 (units)	S_1_ = 0 andD = 0 (units)
Tone as S_1_or D	7	20	0	35
Noise as S_1_or D	45	16	1	0

**Figure 4.**
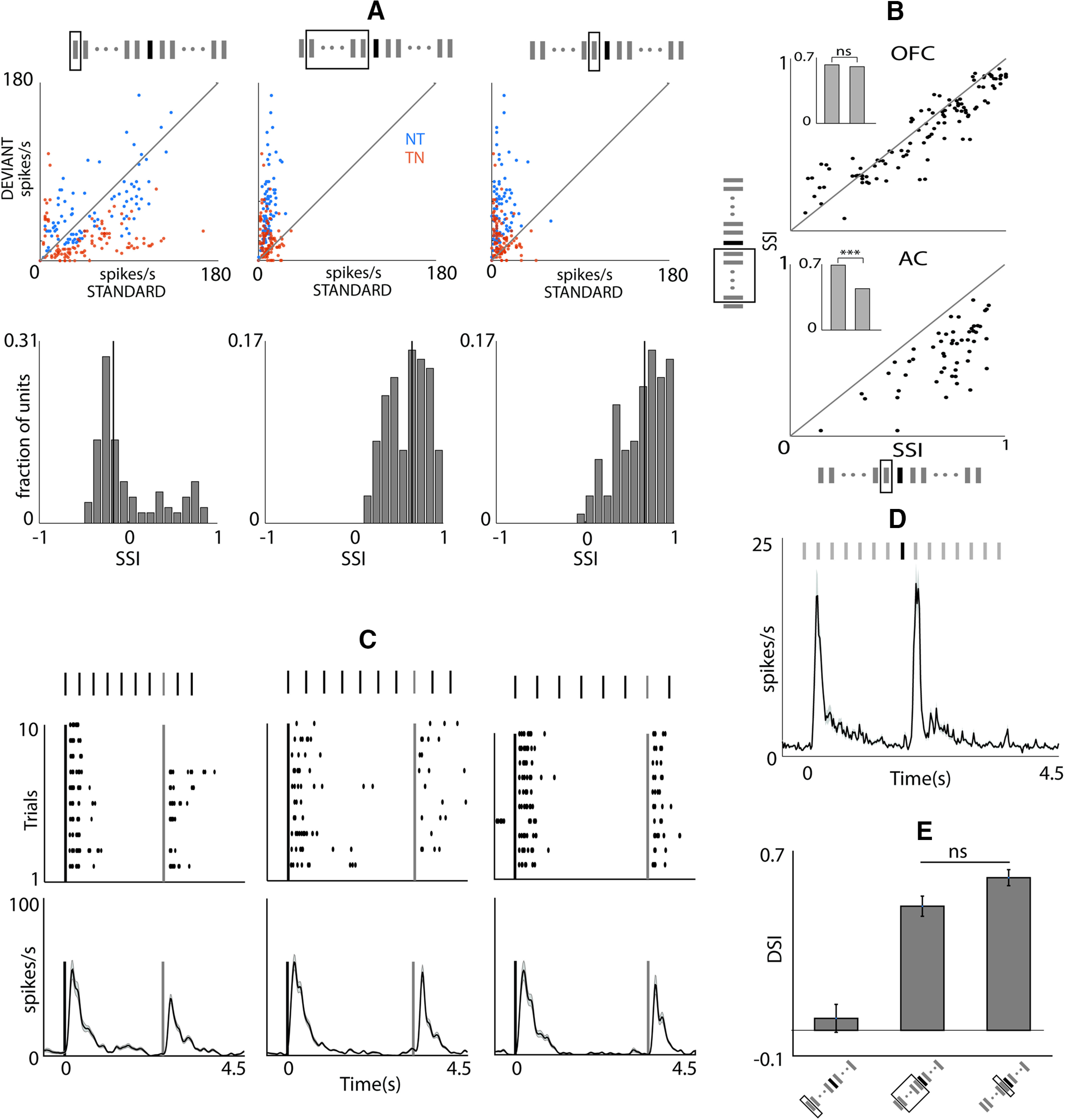
Pure oddball Detection in OFC unlike AC (ii). ***A***, top, Scatter plot of mean spike rate at standard and deviant in NT (blue) and TN (orange). The spike rates for standard were calculated either considering the S_1_ (left), or S_ALL_ (middle), or S_PT_ (right). The tokens considered in the two cases are enclosed in the gray rectangle. The histograms at the bottom show the CSIs computed by using three different standards as described in ***A***. ***B***, Scatter plot of CSIs in OFC (top) and AC (bottom) calculated by taking S_ALL_ and S_PT_. The mean CSIs in the two cases are shown in the bar plots in the inset. ***C***, top, Raster plots of an example unit in response to oddball stimulus with different IToI; 300 ms (left), 400 ms (middle), and 500 ms (right). Bottom, Mean population PSTH for these intervals. ***D***, Mean population PSTH to tone-tone oddball stimulus. ***E***, Mean DSI for tone-tone oddball stimulus with three different standards as described in ***A***. ns, not significant, ***p < 0.001.

To find the neuron’s inherent preference to the deviant either T or N, we calculated CSI in three different ways based on the choice of S tokens considered for S_X_ and S_XS_ in Equation 2. We compared the spike rates in response to either the S_1_ and D or all standard tokens (S_ALL_) preceding D except S_1_ or just the previous token (S_P_) to D. Comparison of responses in scatter plots (Fig. 4*A*) of spikes rates of S_1_ and D (left), S_ALL_ and D (middle) and S_P_ and D (right) for NT and TN (blue and orange, respectively) shows lack of responses to S beyond S_1_. Further, the different CSI distributions (Fig. 4*A*, bottom) show that the OFC neurons respond strongly to S_1_ as compared with D probably because S_1_ is a bigger change than D in the stimulus space. The scatter plots of spike rates and CSI indices of S_ALL_ and D, and S_P_ and D look very similar, suggesting that OFC neurons do not respond to any S except S_1_. The CSI distributions calculated by taking either S_ALL_ and D or S_P_ and D, were not significantly different in the OFC, whereas in AC these two distributions were significantly different (*p* < 0.001, *t* test; Fig. 4*B*). These results indicate a strong adaptation onset right from the second token (beginning of repetition) in OFC, unlike AC suggesting a hierarchy of SSA strength along this pathway.

One of the possible reasons that OFC neurons do not respond to any of the S tokens following the response to S_1_ could be faster rate of stimulus presentation. So instead of the 200 (usually)/250 ms intertoken interval (IToI), we also considered IToIs of 300 (27 units), 400 (41 units), and 500 ms (40 units). Single neuron raster plots and population mean PSTHs of responses to oddball stimuli (Fig. 4*C*) showed no responses to any of the sound tokens other than S_1_ and D, again showing pure oddball detection like responses.

To consider the generality of the pure oddball detection with two narrowband sounds instead of one narrowband and one broadband, we also collected responses to oddball stimuli with two tones (TT; 102 units) but not necessarily in pairs as in the NT/TN cases. As with TN and NT cases, there was a lack of responses to the S token beyond S_1_ and a strong response to the D tone (Fig. 4*D*). Comparing the mean DSI (Fig. 4*E*) among the three ways of computing selectivity index (considering S_X_ in Eq. 3, to be the response to S_1_, S_ALL_, and S_P_) showed no significant difference between DSI_ALL_ and DSI_P_ (0.49 and 0.6, NS, ANOVA). In a small subset of units (*n *=* *25), for which paired data were available with the same stimulus parameters (repetition rate, position of D) as the TN and NT dataset, CSI_ALL_ and CSI_P_ were also not different (0.23 and 0.26, NS, ANOVA). The comparatively lower CSI value could be because of lack of responses to some of the tones as D in this dataset or higher spontaneous rate, which also further shows strong adaptation to the S tone. Thus, OFC neurons probed with oddball stimuli show that they inherently detect changes or violations to the regularity in the stimulus space and could be an important attribute required for flexible value updating in the OFC.

### Sparse responses in the OFC of awake, passively listening mice also show oddball detection

**Figure 5.**
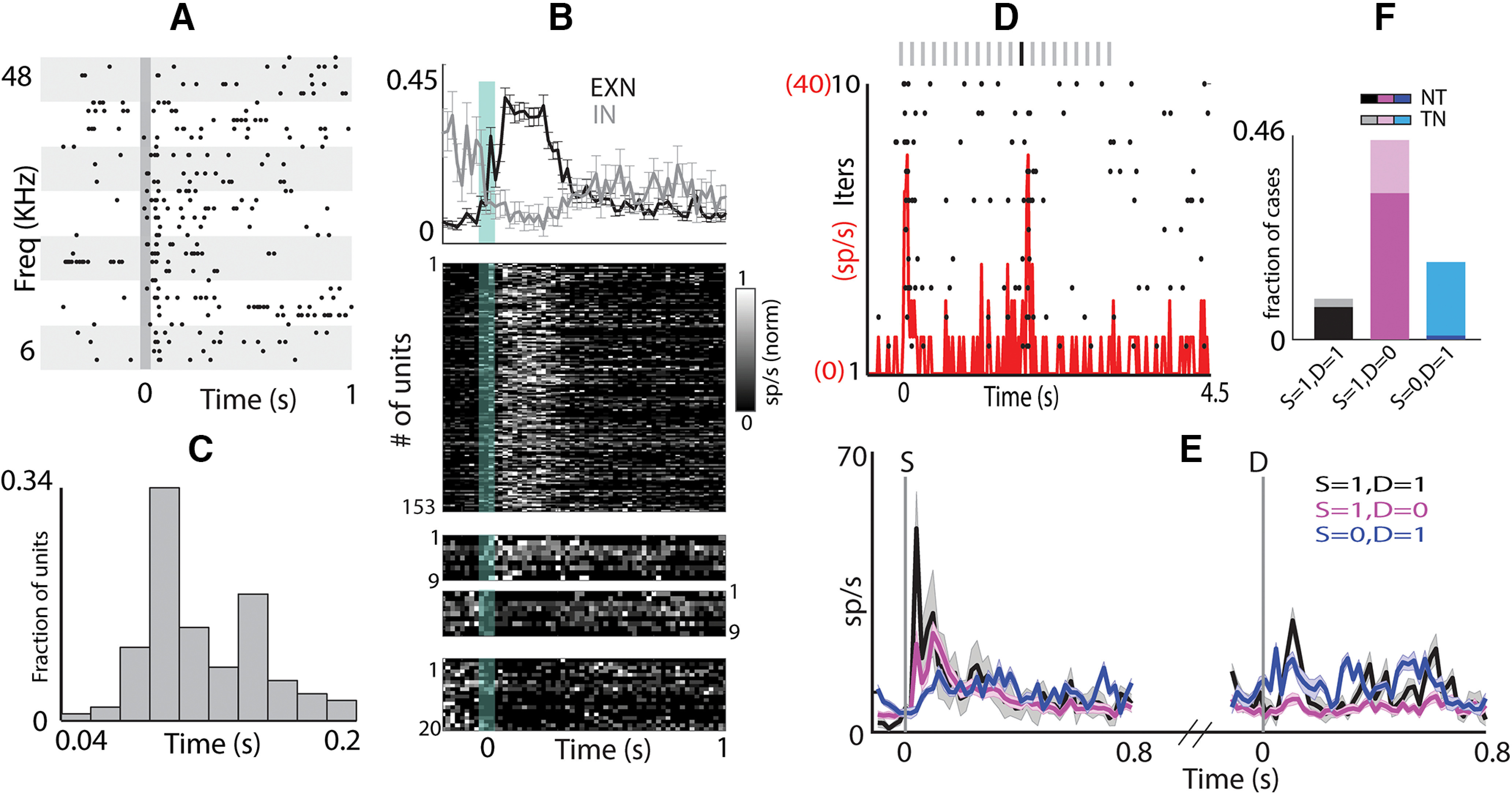
Awake mouse OFC also shows deviance detection. ***A***, Raster plot of an example unit in the OFC showing responses to tones of different frequencies in awake condition. ***B***, top, Mean normalized PSTH ± SEM of all units showing excitation (black) and inhibition (gray) on auditory stimulation. Bottom, Individual unit’s PSTH (upper panel: units showing excitation; middle two panels: units showing excitation to some frequency and inhibition to some other frequency; lower panel: units showing inhibition). ***C***, A distribution of peak response latency in the awake condition. ***D***, Raster plot (black) and PSTH (red) of an example unit showing responses to oddball stimulus. ***E***, Mean population PSTH ± SEM of units responding to both standard onset and deviant (S = 1, D = 1; black), responding only to standard onset (S = 1, D = 0; magenta) and responding only to deviant (S = 0, D = 1; blue). ***F***, Fraction of units belonging to different categories as described in ***E***. The darker shades show the fraction of units belonging to NT and lighter shades show fraction of units belonging to TN group.

To confirm that the observed deviance detection in the OFC is also present in neurons in awake condition, single-unit recordings in passively listening mice were performed with electrodes implanted in the left OFC. As in the anesthetized case, we found robust responses to auditory stimulation, with 73% (162/219) units showing excitatory responses to pure tones (Fig. 5*A*,*B*), which is a much higher proportion of neurons than in an earlier study (Winkowski et al., 2018). The neurons showed expectedly much shorter latency to peak (134.5 ± 2.83 ms SEM; Fig. 5*C*) than in the anesthetized mice, while a small percentage (14%) of units showed a very short latency (∼14 ms) and were not included in further analyses as they could be related to sound-evoked movement. Similar to anesthetized mice, a small fraction of neurons (4%) got suppressed on presentation of tones in the awake state as well.

In the awake head-fixed passively listening mice, responses to TN and NT pairs of oddball stimuli were collected from 54 units (108 cases; Fig. 5*D*,*E*) which responded to at least one of the four stimuli (two S_1_ tokens and two D tokens), as earlier. The population PSTHs (Fig. 5*E*) shows that neurons responded only to the S_1_ and then to the D token and not to the other tokens, as was observed in the anesthetized animals. Thus, the basic observation of immediate adaptation to a S token and response only to the D token, if at all, giving rise to deviant selectivity, is the same as in the anesthetized mice. However, the responses in the oddball case were far more selective, sparse and context dependent in the case of passively listening mice than in anesthetized mice. In 30/108 (28%) cases, there were responses to neither S_1_ nor D, which is significantly larger than that in the anesthetized OFC and AC (OFC: 34/218, *p* < 0.01 and AC: 12/124, *p* < 0.001, two proportion *z* test). In 10/108 cases, there were responses to both S_1_ and D (eight NT and two TN), 49/108 cases to only S_1_ (36 NT and 13 TN), and 19/108 cases to only the D token (1 NT and 18 TN; Fig. 5*F*). Thus, overall, in the awake condition, there were responses to the D token in 27% (29/108) cases as compared with 53% cases in the anesthetized OFC (Fig. 3*B*), showing even higher selectivity of responses. When considering the same sound tokens as S and D, unlike the anesthetized case, we found far stronger context dependence of responses, particularly to tones. In 1(14), 14(30), 8(6), and 31(4) out of 54 units, there were responses to tones (noise), both as S_1_ and D, only as S_1_ and not as D, only as D and not as S_1_, and to neither S_1_ nor as D, respectively. Thus, the responses to tones were far sparser and selective in the oddball stimuli with only 43% (23/54) units showing responses to the tone (either as S_1_ or as D), although for pure tones there were responses in 73% units (*p* < 0.001, two proportion *z* test).

### OFC receives the major projections from the dorsal AC but the major excitatory drive from the ventral AC

**Figure 6.**
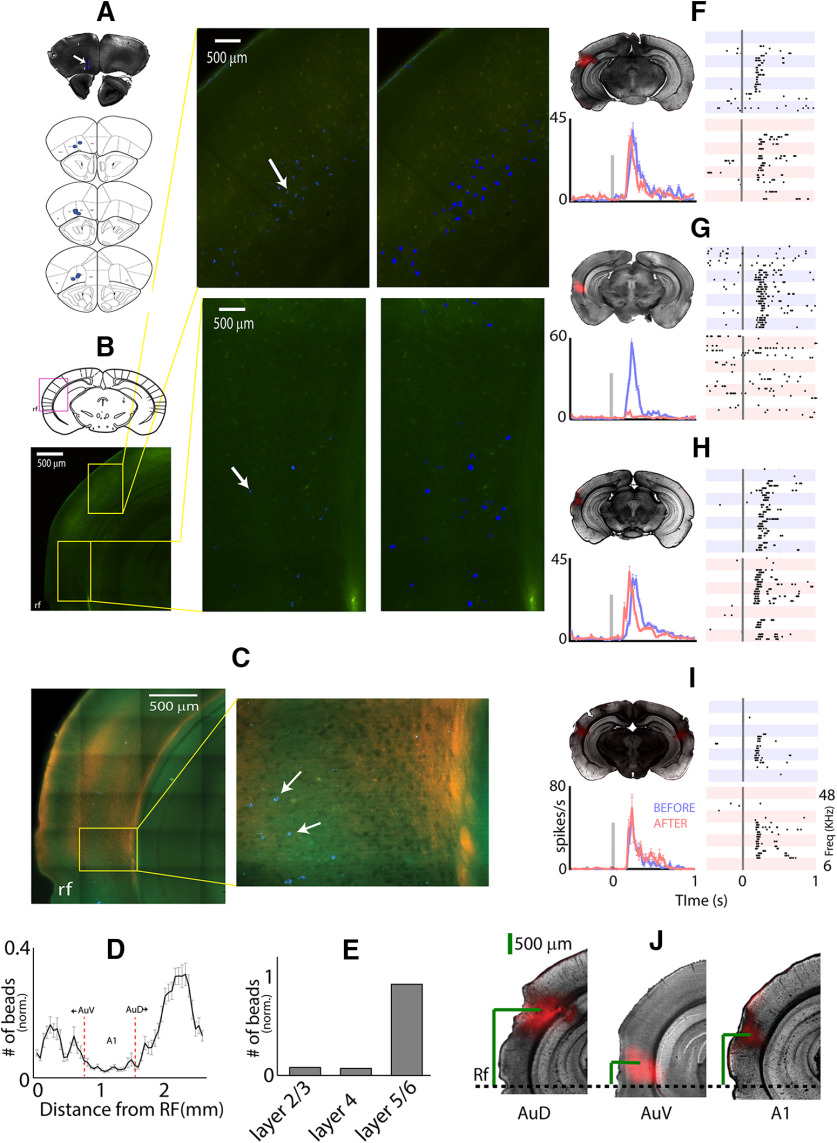
Distinct contributions of AC divisions in OFC auditory responses. ***A***, top, Coronal brain section showing retrobeads injection site (white arrow) in the OFC. Bottom: Injection sites in all nine animals. ***B***, top left, Coronal brain section from mouse atlas showing AC. Magenta box roughly marks the part of the brain region shown in bottom left image. Bottom left, Regions of AuD and AuV are marked in the yellow box. rf: rhinal fissure. Middle, AuD (top) and AuV (bottom) showing labeled cell bodies (white arrows) by the retrograde transported beads from OFC. Right, Same regions with enhanced intensities of the blue pixels for easy visualization of the beads. ***C***, left, Brain section showing layer 4 of the AC labeled with AAV-mcherry injected in the MGBv. Right, Zoomed-in image of the area inside the yellow box on the left showing beads. ***D***, Mean number of beads as a function of distance from the rf. Dashed red lines mark the extent of A1. ***E***, Laminar distribution of beads in the AC. ***F***, Brain section showing block site in the AuD; raster plots of an example unit before and after silencing the AuD; mean population PSTH ± SEM before (blue) and after (red) silencing the AuD. Vertical gray line is the stimulus time. ***G***, Brain section showing block site in the AuV; raster plots of an example unit before and after silencing the AuV; mean population PSTH ± SEM before (blue) and after (red) silencing the AuV. ***H***, Brain section showing block site in the A1; raster plots of an example unit before and after silencing the A1; mean population PSTH ± SEM before (blue) and after (red) silencing the A1. ***I***, Brain section showing dual block sites in both A1; raster plots of an example unit before and after silencing both the A1s; mean population PSTH ± SEM before (blue) and after (red) silencing both the A1s. ***J***, Block sites in the AuD, AuV and A1 from the rf.

To find the main source of auditory afferents in the mice OFC capable of driving auditory responses, we performed neuroanatomical experiments by injecting 200-nl green retrobeads (Lumafluor Inc) stereotactically in the OFC. We specifically targeted the location where we performed electrophysiological recordings (*n *=* *9; Figs. 1*A*, 6*A*). The number of beads in the regions across medio-lateral (ML) extent encompassing the AuV, A1, and AuD were observed and quantified (Fig. 6*B*; Materials and Methods). In a subset of experiments, we injected anterograde *AAV.CB7.CI.mCherry* in MGBv that allowed us to confirm the extent of A1 and AuV along the ML extent. Since both AuV and A1 (Fig. 6*C*) receive MGBv projections (Ohga et al., 2018), the unlabeled region, dorsal to A1, could be identified as AuD. The regions were demarcated in other mice (without MGBv-labeled projections) based on the mouse atlas (Paxinos and Franklin, 2013), which corresponded well with our observations in mice with MGBv injections. Additionally, labeled MGBv projections also allowed us to corroborate lamina specific distribution of cells projecting to the OFC. The average projection profile across the ML extent of AC showed that AuD had the highest density of cells projecting to the OFC, followed by AuV, with minimal projections from A1 (Fig. 6*D*). The lamina specific distributions (Fig. 6*E*) showed that most of the projections to OFC from the AC originated in the infragranular layers (Layer V/VI). Thus, with both A1/AuV and AuD projecting to the OFC, it is likely that both the lemniscal and non-lemniscal pathways are involved in shaping auditory responses in the OFC.

Next, we tested the functionality of projections from AC to the OFC to determine the contribution of the different regions of AC to the auditory responses in the OFC. In a series of experiments, single-unit recordings with responses to pure tones in the OFC were performed before and after pharmacological inactivation of AuD (*n *=* *3), AuV (*n *=* *5), and A1 (*n *=* *7) with muscimol and baclofen (Materials and Methods). The site of inactivation was confirmed by tracking fluorescent SR101 (Fig. 6*F–H*, left top) mixed with the muscimol-baclofen solution.

Contrary to expectation, the inactivation of AuD (Fig. 6*F*) did not affect the single-unit OFC responses to auditory stimulation (Fig. 6*F*, right, example single-unit dot raster). The population mean PSTHs (*n *=* *45 units) before and after AuD inactivation (Fig. 6*F*, bottom left), showed no difference (blue and red, mean spike rates 72.2 ± 3.7 and 65.7 ± 4.8, NS). Significant responses to all frequencies before inactivation were included in constructing the population PSTHs. While the AuV, with a lesser number of neurons than AuD projecting directly to the OFC [0.11 ± 0.02 and 0.2 ± 0.03 beads (normalized), *p* < 0.01 *t* test], was found to be the source of almost the entire auditory-driven excitatory activity in the OFC. Similar plots as before, of example dot raster and population mean PSTHs (*n *=* *43 units) for before and after AuV inactivation (Fig. 6*G*) show that OFC auditory responses were almost completely abolished following AuV block (98.7 ± 3.3 and 14.9 ± 1.4, *p* < 10^−76^). Of course, other than the direct inputs to OFC from AuV, other inputs to OFC providing such excitatory auditory inputs cannot be ruled out, but such indirect pathways also must originate from the AuV. Thus, the dorsal and ventral divisions of non-A1 are in stark contrast of each other in terms of their contribution to OFC auditory responses. However, their direct anatomic connections show characteristics opposite to their functional contributions. Similar to AuD block, the inactivation of A1 (Fig. 6*H*) did not lead to any change in firing rates (70.1 ± 2.4 and 72.9 ± 3.4, NS), as observed in population mean PSTHs (*n *=* *62 units) and dot raster plots (Fig. 6*H*). The possibility of a contralateral A1 contribution through indirect pathways to OFC was also considered. Bilateral inactivation of A1 (Fig. 6*I*, top left) also did not change firing rates of OFC neurons as assessed through population mean PSTHs (*n *=* *30 units) and mean firing rates of single units in response to tones before and after pharmacological inactivation of both A1s (61.7 ± 10.07 and 79.5 ± 11.3, NS).

### Auditory responses in the OFC originate from both, the lemniscal and non-lemniscal MGB

**Figure 7.**
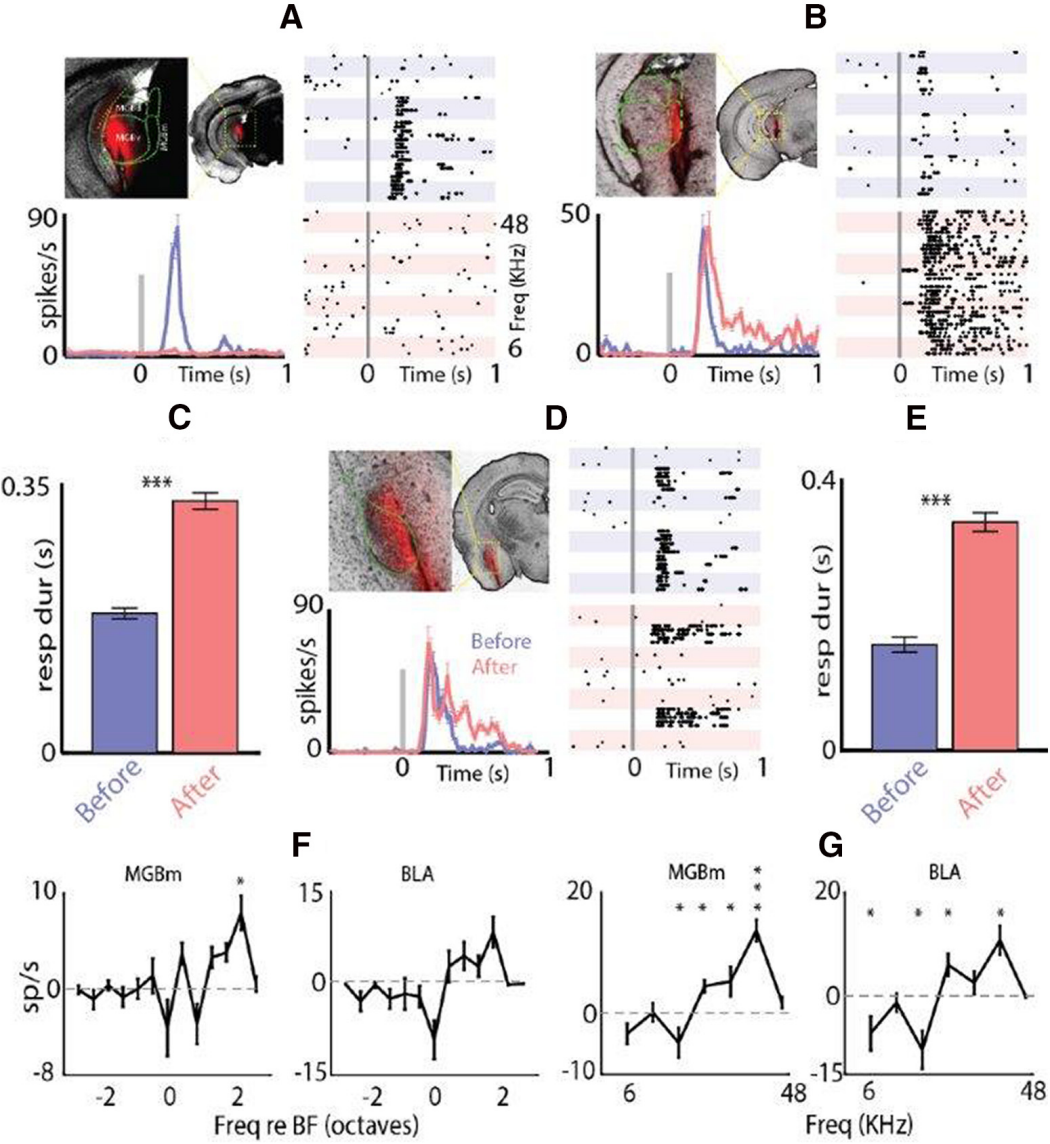
Parallel excitatory and inhibitory contributions to OFC auditory responses originate in the lemniscal and non-lemniscal auditory thalamic nuclei. ***A***, Brain section showing block site in the MGBv; raster plots of an example unit before and after silencing the MGBv; mean population PSTH ± SEM before (blue) and after (red) silencing MGBv. Vertical gray line is the stimulus time. ***B***, Brain section showing block site in the MGBm; raster plots of an example unit before and after silencing the MGBm; mean population PSTH ± SEM before (blue) and after (red) silencing the MGBm. ***C***, Mean response duration before and after silencing the MGBm. ***D***, Brain section showing block site in the BLA; raster plots of an example unit before and after silencing the BLA; mean population PSTH ± SEM before (blue) and after (red) silencing the BLA. ***E***, Mean response duration before and after silencing the BLA. Mean inhibitory inputs into OFC, from MGBm and BLA as a function of (***F***) frequency with respect to BF and (***G***) absolute frequency. *p < 0.05, ***p < 0.001.

Since A1 and AuD inactivation did not cause changes in response rates in the OFC and given that MGBv projects on AuV as well (Ohga et al., 2018), we hypothesized that OFC auditory responses originate in the lemniscal MGBv. To test the hypothesis, single-unit recordings in response to pure tones in the OFC were performed before and after MGBv inactivation (Fig. 7*A*). As with AuV inactivation, auditory responses in the OFC were completely abolished with MGBv block (23 units, 121.3 ± 9.7 and 18.5 ± 1.5, *p* < 10^−16^). Since MGBv efferents almost entirely project to A1/AAF and AuV, we conclude that the entire auditory driven excitatory input originates in the lemniscal auditory thalamus (MGBv). For our MGBv inactivation, we confirmed *post hoc* (Fig. 7*A*, top left, inset) the lack of SR101 in dorsal MGB (MGBd; Paxinos and Franklin, 2013) to rule out the inactivation of the neurons projecting to AuV from there. Physical damage to MGBd during GABA agonist injections to MGBv was also ruled out by repeating the experiments with saline injections to MGBv which did not alter tone response rates in the OFC (*n *=* *18 units, 93 ± 6 and 75.6 ± 6.6, NS). Also, since AuD with major thalamic afferents from MGBd (Honma et al., 2013; Kok and Lomber, 2017) did not alter OFC responses, the involvement of the non-lemniscal MGBd in OFC auditory responses are at best minimal. The contribution of the MGBm to OFC auditory responses was also tested similarly (Fig. 7*B*). As opposed to abolition or no change in response rates observed in the other inactivation experiments, responses to pure tones altered dramatically in the OFC following inactivation of MGBm. Most units showed a behavior similar to the example single-unit activity before (blue) and after (red) MGBm block shown in Figure 7*B*, right. The population mean PSTHs (42 units) showed no change in peak response rates (54.7 ± 2.8 and 52 ± 2.8, NS, *t* test) with MGBm inactivation, but the responses became persistent, with spiking continuing following the stimulus onset sometimes up to 1 s and mean response duration almost doubling (177 ± 6 and 314 ± 11 ms, *p* < 0.001; Fig. 7*C*). Thus, MGBm, which has multiple sources of auditory inputs as well as other sensory inputs, is a source of long-lasting auditory driven inhibition in the OFC affecting the temporal profile of auditory responses.

Given the strong connection from MGBm to LA (Woodson et al., 2000) and LA to BLA (Ledoux, 2000) and dense projections from BLA to OFC (Lichtenberg et al., 2017), we hypothesized that the major MGBm contribution to OFC auditory responses is through the BLA and the effects of BLA inactivation would produce an effect similar to that of MGBm silencing. As expected, on inactivating the BLA (Fig. 7*D*), we found a similar response pattern with long-lasting persistent activity in the OFC (Fig. 7*D*, right, example dot raster), with no change in peak firing rates as assessed through the population mean PSTHs before and after BLA block (56 units, 68.7 ± 4.6 and 78.9 ± 5, NS, *t* test). As with MGBm block, response duration increased (Fig. 7*E*) by similar degrees with BLA inactivation (response duration: 156 ± 11 and 338 ± 14, *p* < 0.001, *t* test).

The change in response to tones after MGBm or BLA inactivation was quantified by considering the difference in mean rate responses (after-before) to the different frequencies (in the window of response duration after inactivation; Materials and Methods) relative to the BF of the units (Fig. 7*F*, MGBm, block left, BLA block, right). In both cases, a similar pattern of rate difference was observed, with almost all frequency components relative to BF, showing no significant difference except a peak at two octaves above BF and a negative peak at BF. Thus, the MGBm or BLA-based inhibition into the OFC is not organized in a BF-specific way. Rather when considering the changes in firing rate before and after MGBm or BLA inactivation (Fig. 7*G*, left and right) in absolute frequency, we found that significant inhibitory inputs are in the middle frequency region of mouse hearing (17–34 kHz). Further, we observed that there is a trend of a significant net excitatory contribution mediated through both MGBm and BLA in the lower frequencies (6–12 kHz). Since the pathway originating in the MGBm is associated with fear conditioning, it is likely that this pathway is shaped based on the previous natural experience of fear associated stimuli and auditory events.

### Auditory response properties and deviant selectivity of OFC: contributions of auditory input sources

**Figure 8.**
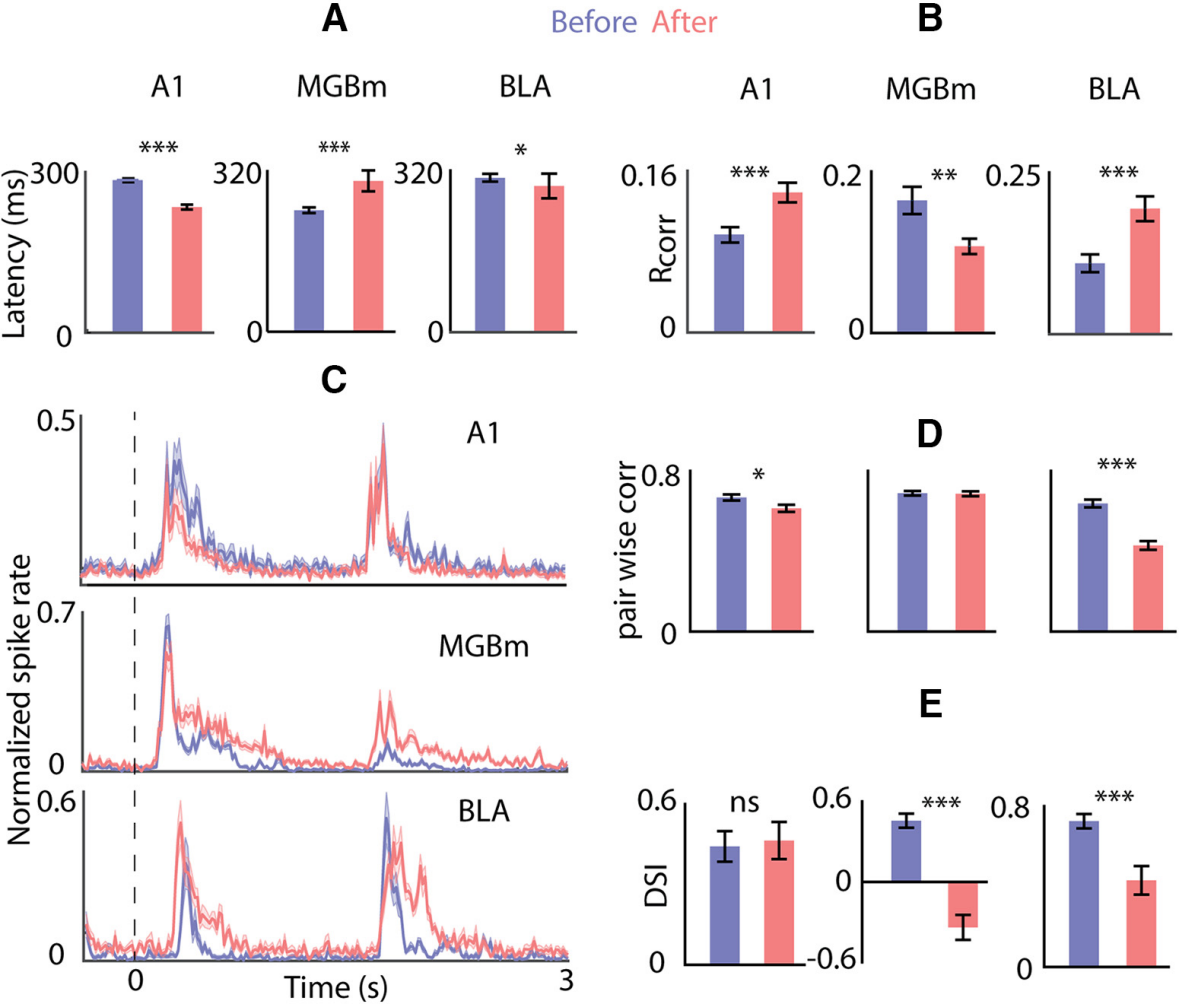
Both OFC deviant selectivity and spike time-based response properties are shaped by the non-lemniscal pathway unlike by A1. ***A***, Mean population latency ± SEM to pure tones before (blue) and after (red) silencing A1 (left), MGBm (middle), and BLA (right). ***B***, Mean population reliability (R_corr_) ± SEM before (blue) and after (red) silencing A1 (left), MGBm (middle), and BLA (right). ***C***, Mean population PSTH ± SEM in response to oddball stimulus before (blue) and after (red) silencing A1 (top), MGBm (middle), and BLA (bottom). ***D***, Mean population pairwise correlations ± SEM before (blue) and after (red) silencing A1 (left), MGBm (middle), and BLA (right). ***E***, Mean population DSI ± SEM before (blue) and after (red) silencing A1 (left), MGBm (middle), and BLA (right). *p < 0.05, **p < 0.01,***p < 0.001.

With the inactivation of A1, although there are no changes in firing rate, there is a significant reduction in the latency of responses to tones in single units of the OFC (306 ± 3.4, 258 ± 4.33 ms, *p* < 0.001, paired *t* test; Fig. 8*A*, left). Such latency reduction is usually associated with, a stimulus getting effectively stronger (for example, with an increasing sound level of noise; Fig. 1*B*), weaker long-term adaptation (Fig. 2*E*), or disinhibition. Similarly, on considering latencies before and after inactivation of MGBm and BLA, opposing effects of latency were found, although in both cases similar changes were observed in terms of response rates and response duration. MGBm inactivation led to a marked increase in latency to tones (246.5 ± 4.4, 296.6 ± 7.3 ms, *p* < 0.001, paired *t* test; Fig. 8*A*, middle) while inactivation of BLA barely led to a reduction in latency (315.4 ± 7.4, 297.4 ± 8 ms, NS *t* test, removing the effect of outliers and comparing medians, *p* < 0.05 rank-sum). Since the initial latency of response varied between the two populations of single units (before MGBm block and before BLA block), we also compared the fractional change in latency of single units on MGBm inactivation and BLA inactivation (mean 27% and 7% unpaired *t* test *p* < 0.001). Thus, the effective inhibition in the OFC, originating from MGBm is not simply relayed by the BLA. There are likely other auditory sources that also provide inhibition via BLA on to OFC. Such sources of inhibition on OFC through BLA, likely from AC (Bertero et al., 2019) remain intact on MGBm inactivation leading to longer latency, while with BLA inactivation their effect is removed to lead to the reduced latency.

Spike timing is crucial in generating synchronization in populations of neurons (Kreuz et al., 2007; Ermentrout et al., 2008), plasticity (Benedetti et al., 2009; Li et al., 2014), and forming associations (Atilgan et al., 2018). We considered spike timing reliability in the OFC single units before and after the inactivation of the different auditory sources. A1 inactivation remarkably increased spike timing reliability quantified through R_corr_ (from 0.0953 ± 0.01 to 0.1385 ± 0.01, *p* < 0.001, paired *t* test; Fig. 8*B*, left) in response to tones. Although the absolute R_corr_ values were low, the increase on block was highly significant. Again, a differential effect was observed with BLA and MGBm inactivation. While MGBm inactivation decreased spike timing reliability (from 0.16 ± 0.02, to 0.11 ± 0.01, *p* < 0.01, paired *t* test; Fig. 8*B*, middle), BLA inactivation nearly doubled reliability of spike times (from 0.11 ± 0.02 to 0.19 ± 0.02, *p* < 0.001, paired *t* test; Fig. 8*B*, right) in the repeated presentation of tones. Since inhibition is crucial in precise spike timing (Pouille and Scanziani, 2001; Wehr and Zador, 2003), and with MGBm having the highest reliability in spiking (Anderson and Linden, 2011) among the auditory thalamic nuclei, the loss of BLA mediated feedforward inhibition originating from MGBm, may lead to the decreased reliability is spiking in the OFC. However, since A1 acts as a source of spike timing jitter in the OFC (Fig. 8*B*, left), blocking of BLA also effectively removes the effect of A1 dominated spiking jitter introduced in the OFC through LA.

As our data suggest that context dependence and pure oddball detection are the hallmark of the auditory responses of the OFC, we used responses to oddball stimuli before and after inactivation to further understand the contribution of the different auditory sources to response properties of the OFC (Fig. 8*C*). Pairwise correlations can strengthen coding efficiency (Hung et al., 2015) and enhance plasticity (Feldman, 2009). We compared synchronization of responses in pairs of simultaneously recorded OFC single units before and after inactivation of each of the auditory sources. Inactivation of A1 produced a small decrease in synchronization with oddball stimuli (from 0.67 ± 0.01 to 0.61 ± 0.02, *p* < 0.05, *t* test; Fig. 8*D*, left). However, MGBm inactivation caused no change in pairwise correlations (0.69 ± 0.01 and 0.69 ± 0.01, NS; Fig. 8*D*, middle), while BLA inactivation reduced pairwise correlations (from 0.64 ± 0.02 to 0.43 ± 0.02, *p* < 0.001 *t* test; Fig. 8*D*, right). Thus, the effective inhibitory input to the OFC from the BLA synchronizes activity across populations of single units, independent of the MGBm input to the BLA. As BLA inactivation increases spike timing reliability of single neurons, it suggests that auditory driven indirect AC input to the OFC via BLA causes synchronization across pairs of neurons. The above is further corroborated by the fact that A1 inactivation reduced pairwise correlations in the OFC, although to a lesser degree.

Finally, we consider the contribution of different auditory sources on the oddball detection property of OFC single units. Since we did not have sufficient data on pairs of NT and TN both before and after inactivation of different structures, we considered DSI instead of CSI to quantify oddball selectivity. Population mean PSTHs of OFC single units in response to oddball stimuli before and after inactivation of A1, MGBm, and BLA (Fig. 8*C*, each row) show the emergence of persistent activity at both the onset and the deviant except in the case of A1 block. Quantification of the selectivity (DSI) before and after block shows that A1 does not contribute to DSI in the OFC, while inactivation of both the MGBm and the BLA produced a drastic reduction in DSI (from 0.44 ± 0.06 to 0.46 ± 0.07, *p* = 0.57, 0.46 ± 0.05 to −0.34 ± 0.09, *p* < 0.001, and 0.72 ± 0.04 to 0.43 ± 0.07, *p* < 0.001, respectively, *t* test; Fig. 8*E*). The difference in the reduction of DSI in case of MGBm and BLA inactivation suggests that the primary source of the selectivity to oddball stimuli in the OFC is the MGBm, while it is further strengthened by auditory inputs from the BLA from sources other than MGBm (like AC) to the OFC. However, it should be noted that with neither MGBm nor BLA inactivation, responses to subsequent tokens after the onset token appeared. Thus, although values of DSI reduce on average to produce lower oddball selectivity in both cases (MGBm and BLA inactivation), the OFC’s intrinsic selectivity to the deviant and fast adaptation to subsequent stimuli over a long timescale is unchanged. AuV, the primary excitatory input to the OFC, itself does not show similar deviant selectivity and adaptation (Fig. 3*D*).

## Discussion

We find the presence of robust auditory responses in single units of the mouse OFC during passive listening both in awake as well as in the anesthetized state. Auditory responses in the OFC as in AC showed robust responses to tones and noise, the usual stimuli used for characterization. Other studies probing the OFC with different kinds of auditory stimuli in mice (Winkowski et al., 2018) and primates (Rolls et al., 2006) have also found robust responses. However, in our study, we show that the OFC auditory responses were drastically different from AC in several major ways which have not been studied in the OFC previously like (1) absence of topographical organization; (2) longer history dependence and adaptation; and (3) presence of pure oddball detection. Although AC auditory responses are also affected by multiple timescales of adaptation (Ulanovsky et al., 2004), OFC neurons showed extremely long history dependence and adaptation lasting above 10 s as observed with comparisons of OFC and A1/AuV responses with a variety of ITIs (Fig. 2). Since the outcome of a stimulus is almost always temporally offset by large durations (Pavlov, 1927; Fuster and Alexander, 1971), this long history dependence in the OFC may potentially encode the sensory aspect of the auditory memory over long timescales and may play a key role in creating various stimulus-outcome associations (Delamater, 2007; Rudebeck et al., 2008; Sadacca et al., 2018) and their revaluation. The OFC neurons show pure oddball detection during oddball stimulus streams, ceasing to respond from the first instant of repetition of the standard stimulus. This degree of faster and stronger adaptation to repetition is not seen in AC and appears to emerge in the OFC possibly achieved by local circuits within OFC through recurrence (Yarden and Nelken, 2017).

In our pharmacological block experiments, we found that A1 does not contribute to OFC response strength but only induces more jitter in spike timing and longer response latency. This effect could be via an early inhibition through a weak input along the A1/AuV-LA-BLA (Romanski et al., 1993; Ledoux, 2000; Tsukano et al., 2019) or directly from A1 to OFC pathways (Fig. 6*D*,*H*). Through anatomic tracing, we found that secondary areas of the AC send most of the projections to OFC within AC with AuD showing the strongest labeling. Despite the strongest labeling, the OFC responses were not affected on inactivating AuD. The mouse AuD is more involved in representing the perceptual meaning of primarily temporally structured sounds while AuV is thought to represent value in terms of novelty (Weible et al., 2014; Geissler et al., 2016). The OFC is involved in stimulus outcome value computation, which is consistent with the result that AuV drives excitatory auditory responses in the OFC. AuD, on the other hand, is likely recruited to provide inputs to the OFC in a more behavioral context-specific manner with ultrasonic complex vocalizations (Tsukano et al., 2015), multimodal stimuli (Morrill and Hasenstaub, 2018), or multimodal spatial tuning (Bizley and King, 2009). The function and necessity of the AuD projections on to the OFC require further investigation. Since OFC responses were abolished on silencing MGBv as with inactivation of AuV which receives direct inputs from the MGBv (Fig. 7*A*; Ohga et al., 2018), we hypothesize that the OFC auditory responses are driven by MGBv via AuV.

We also show that auditory inputs to the OFC originate in at least two parallel regions in the MGB, the ventral and medial divisions. These two streams converge in at least two locations, the amygdala, and the OFC. The inputs from AuV carry in sensory information with context dependence which is sharpened through the long-lasting deviant selective inhibitory drive originating from the MGBm and modified in the BLA and also through local recurrent connections in the OFC. Both MGBm and BLA could provide a saliency filtering of the sensory inputs to the OFC and suppress responses in OFC units following deviant/salient auditory events. The MGBm and BLA also provide a means of controlling the sensory driven activity by causing persistent activity in the OFC which may allow the sensory stimulus to be associated with other outcome related delayed signals related to reward (Thorpe et al., 1983; Hikosaka and Watanabe, 2000; Stalnaker et al., 2014), prediction error (O'Neill and Schultz, 2013), or punishment (O'Doherty et al., 2001; Windmann et al., 2006) required for reinforcement in acquisition as well as in reversal learning. Such longer persistent activity is seen in the PFC during working memory related behavior (Fuster and Alexander, 1971; Funahashi et al., 1989; Curtis and D'Esposito, 2003) and also in the OFC conveying incentive value of cues (Gallagher et al., 1999; Tremblay and Schultz, 1999). A study showed the presence of a group of neurons in the MGBm that silences itself during conditioned stimulus presentation (O'Connor et al., 1997). MGBm is the first station in the auditory pathway that shows changes in the firing rate because of task-related stimulus associations (Birt et al., 1979) and forms part of the thalamo-amygdaloid component in the auditory pathway (Ledoux et al., 1986) crucial in fear learning. We also show that frequency profile of the inhibitory inputs from the MGBm via the LA-BLA-OFC pathway is innately tuned to the 30-kHz region (Fig. 7*G*), which is also the frequency range of fear-induced vocalizations. Thus, the MGBm driven control of OFC activity is likely to do with associations of fear eliciting stimuli (Weinberger et al., 1995; Weinberger, 2011).

Silencing BLA causes decorrelation of response in pairs of OFC units and increase in spike timing precision across trials, other than causing persistence and lowering of deviant detection. Precisely timed spiking in the persistent OFC activity may aid in plasticity (Markram et al., 1997; Bi and Poo, 1998; Dan and Poo, 2004; Feldman, 2009) required for associating the auditory stimulus with delayed outcome related signals. Similarly, decorrelation of activity, removing redundancies in the population, allows more possibilities of creating associations with the OFC stimulus driven activity and outcome signals. BLA is known to encode valence (Paton et al., 2006; Janak and Tye, 2015; Zhang and Li, 2018) and damage to BLA-OFC connections may lead to disruption of decision-making and reversal learning like behaviors (Zeeb and Winstanley, 2013; Orsini et al., 2015; Lichtenberg et al., 2017; Groman et al., 2019). We hypothesize that these disruptions may arise because of lack of deviant selectivity and precise inhibitory control imposed by the BLA affecting the required persistent activity. Thus, we propose that the MGBm via LA-BLA and BLA itself act as controllers of persistent activity required for stimulus-outcome associations during behaviors like decision-making and reversal learning.

A limitation of our study is that the experiments revealing the contributions of different auditory areas to the OFC responses were conducted in the anesthetized animals. Hence the behavioral significance of these findings could not be established. Besides, the effects of indirect pathways to the OFC that may get affected on silencing an area cannot be ruled out with the current experiments.

## Synthesis

Reviewing Editor: Christine Portfors, Washington State University Decisions are customarily a result of the Reviewing Editor and the peer reviewers coming together and discussing their recommendations until a consensus is reached. When revisions are invited, a fact-based synthesis statement explaining their decision and outlining what is needed to prepare a revision will be listed below. Note: If this manuscript was transferred from JNeurosci and a decision was made to accept the manuscript without peer review, a brief statement to this effect will instead be what is listed below.

Synthesis:

The reviewers think that the data are potentially very interesting, but the manuscript is still challenging to read. The reviewers do not think the comments from the previous reviewers for the Journal of Neuroscience submission were adequately addressed. There are still specific concerns outlined by Reviewer 1. A major concern is the length of the manuscript, its organization, and the quality of the writing. The manuscript would benefit a lot if the authors could ask some colleagues, experts in the field and ideally native English speaking to read and make comments of their manuscript.

Reviewer 1 Comments:

I have already reviewed this manuscript for the Journal of neuroscience. While I appreciate the work made by the authors in this new revision, unfortunately, some of my principal comments remain unsolved. For example, I commented that the manuscript is long and verbose. And this still applies, for example, the beginning of results is totally unnecessary. In my view this is like an introduction, and in fact much of it should be moved to introduction. Moreover, authors need to make clear what is similar and what is new in their results to Winkowski et al., 2017 (Apologies, I meant to mention that the principal author was a coauthor with previous work in 2013).

Although I appreciate the efforts made by the authors to clarify the oddball paradigm it is still unclear to me. I think that since they use a variant of the classical oddball paradigm an illustration is needed to show the stimulus parameters etc. BTW, oddball is a single word... authors used 2 words: odd ball, a single word.... or even a third possibility: two words with a hyphen .... I think this is incorrect, at least in the literature oddball is usually referred to as a single word. Please change throughout MS.

Authors study the topography of BF along the horizontal plane, have they consider other dimensions like depth and the lateromedial axis?

Again, the ref to Antunes or Ulanovsky and colleagues is unnecessary in results...and also the sentence is a not grammatically elegant.... While stimulus presentation rates in the auditory pathway, to rule out any effect 322 of adaptation, have been long established (Antunes et al., 2010),.....

The section on Neurons in the OFC show high context-dependence and pure oddball detection unlike in the AC is difficult to follow, authors should really try to simply this section.

In my previous review I also commented on the use of deviance detection. Authors claim in the rebuttal letter that they don´t used it now, but there is a heading that included deviance detection.... Sparse responses in the OFC of awake, passively listening mice also show deviance detection. So, authors should be more systematic and also simplify the concepts. They now talk about ‘pure oddball detection’ This must be defined, and again there is no point to create new terms if there is not a functional meaning.

There must be a misunderstanding about my previous comment on deactivation. Indeed, this is a standard neurophysiological technique, although there are others which are also good or perhaps even better like cooling or microiontophoretic injection that allow a reverse effect. What I meant was if authors deactivate for example inhibitory neurons, this could lead to an excitation as a result of a disinhibitory effect.

In discussions authors state that: However, in our study, we show that the OFC auditory responses were drastically different from AC responses in several major ways, which have not been studied in the OFC previously. Here it would be good if they enumerate one by one, so the reader gets a clear picture of these differences.

The discussion is still long and very speculative. Perhaps authors can look at two recent papers that may be interesting in relation to their findings since they mention about reward prediction errors

bioRxiv: https://www.biorxiv.org/content/10.1101/2020.04.14.041152v1.full.pdf

and

bioRxiv: https://www.biorxiv.org/content/10.1101/2020.02.17.953141v1.full.pdf

But of course, I appreciate this final suggestion is a reviewer´s opinion that may be out of the scope of the authors.
